# Multidrug Resistance: Are We Still Afraid of the Big Bad Wolf

**DOI:** 10.3390/ph18060895

**Published:** 2025-06-14

**Authors:** Abdulelah Alhazza, Adenike Oyegbesan, Emira Bousoik, Hamidreza Montazeri Aliabadi

**Affiliations:** 1 Department of Biomedical and Pharmaceutical Sciences, Chapman University School of Pharmacy, Harry and Diane Rinker Health Science Campus, #211, 9401 Jeronimo Road, Irvine, CA 92618, USA; 2 Department of Pharmaceutics, Faculty of Pharmacy, Northern Border University, Rafha 76313, Saudi Arabia; 3 Faculty of Pharmacy, University of Derna, Al-Fataeh Campus, Derna 21881, Libya

**Keywords:** multidrug resistance, acquired resistance, intrinsic resistance, cancer, chemotherapy, molecularly targeted drugs

## Abstract

After the era of multidrug resistance (MDR) against cytotoxic chemotherapy, the development of resistance against newly developed molecularly targeted drugs also seems inevitable. While the mechanisms involved in resistance against these two categories of anticancer drugs are different, the principles are similar: inherent resistance (also known as primary resistance) is a result of heterogeneity in cancer cells where a subpopulation of the cells do not show a favorable initial response to the drug, while acquired resistance (or secondary resistance), as the name suggests, is developed after repeated treatments due to the plasticity of cancer cells. Despite the introduction of a variety of molecularly targeted drugs to clinical practice, chemotherapy is still at the forefront of the battle against cancer. In this manuscript, we review the major mechanisms involved in MDR and resistance against different categories of molecularly targeted drugs separately, and review some of the strategies studied to overcome the resistance against cancer therapy. While MDR mechanisms have been reviewed previously, the molecular mechanisms of resistance to the latest generations of anticancer drugs are rarely reviewed as a group, and the connection between the two categories of resistance is often missing in this type of publication. Our aim is to illustrate a comprehensive picture of what the landscape of cancer treatment is today with respect to resistance. While this picture seems bleak, and it is the common belief that resistance is inevitable, understanding the mechanisms involved could potentially lead to more efficient approaches to overcoming this so far unbeatable obstacle.

## 1. Introduction

Multidrug resistance (MDR) is known as reduced or lack of response to the therapeutic action of anticancer drugs that are structurally and mechanistically unrelated. MDR is to this day a significant challenge in chemotherapy, often leading to a less-than-optimal response, relapse, and mortality. The mechanisms underlying MDR are complicated and in some cases not completely understood, with the exception of the mechanism involved in the enhanced expression of ATP-binding cassette (ABC) transporters [1,2]. Acquired and inherent MDR—the latter also known as intrinsic—have been extensively studied for over 50 years [3,4]. Key early discoveries include the identification of membrane transporters that confer resistance to multiple drugs [5]. Researchers later identified P-glycoprotein (P-gp) as one such transporter, which was later referred to as multidrug resistance protein 1 (MDR1) or ATP-binding cassette subfamily B member 1 (ABCB1) [6,7]. Since then, P-gp has been the most studied transporter and a notable MDR mechanism. Subsequent studies revealed other members of this family of transporters, e.g., breast cancer resistance protein (BCRP/ABCG2) and multidrug resistance-associated protein 1 (MRP1/ABCC1), which have expanded our understanding of the cell repertoire of transporter proteins [7,8,9,10,11].

However, MDR is not restricted to membrane transporter mechanisms. It includes noncellular mechanisms (e.g., geometric resistance of tumor vasculature [12] and elevated interstitial pressure in the tumor microenvironment) and more specific cellular mechanisms that range from inhibition of apoptosis to DNA damage repair [4]. Among these cellular mechanisms, DNA damage repair, topoisomerase mutations, glutathione S-transferase, and modifications in apoptosis mechanisms are more commonly studied and will be discussed in this manuscript.

A new, promising era in cancer therapy began in the late 1990s with the introduction of molecularly targeted drugs, small molecules or antibodies targeting a specific protein essential for cancer progression [13]. However, despite the initial promising response, an unresponsive subpopulation that survives the initial dose and/or acquires resistance will develop with repeated exposure [14]. To date, chemotherapy alone or in combination with molecularly targeted drugs remains the first line of treatment for many types of cancer. Unlike MDR, resistance to molecularly targeted drugs does not typically extend to other drugs, as it arises from more specific molecular mechanisms and is most often related to the structure or the expression levels of the targeted protein, and/or other proteins that compensate for its loss of function. If these mechanisms are active in the cancer cells before drug exposure, they are referred to as exhibiting inherent resistance, reflecting the heterogeneity of cancer cells within a single tumor [15,16]. However, if the mutations leading to structural modifications in protein structure or changes in expression levels are due to exposure to the molecularly targeted drugs, the resistance is termed “acquired.” In this review, we will discuss MDR against chemotherapy in general, as well as against molecularly targeted drugs.

## 2. Methodology

This review was conducted using a narrative approach to analyze and discuss the key mechanisms of multidrug resistance (MDR) and resistance to molecularly targeted therapies in cancer, as well as to review recent strategies proposed to overcome these types of resistance. A comprehensive search was performed in major electronic databases, including PubMed, Scopus, and Google Scholar, using relevant keywords such as “multidrug resistance,” “ABC transporters,” “overcome MDR,” “nanotechnology in cancer therapy,” “chemotherapy resistance,” “targeted therapy,” “molecular targeting,” “apoptosis,” “resistance mechanisms,” “combination therapies,” and “DNA damage response.”

The review includes English-language publications mostly published between 2000 and early 2025; however, key citations dating back to 1960 were also included. References were selected based on their scientific relevance and alignment with the objectives of the review, with a focus on high-quality peer-reviewed articles and recent clinical and experimental studies. The selection prioritized studies that provide insights into molecular and cellular resistance mechanisms and contribute to the development of new therapeutic strategies.

## 3. Mechanisms Contributing to MDR

### 3.1. Transporters

**Figure 1:**
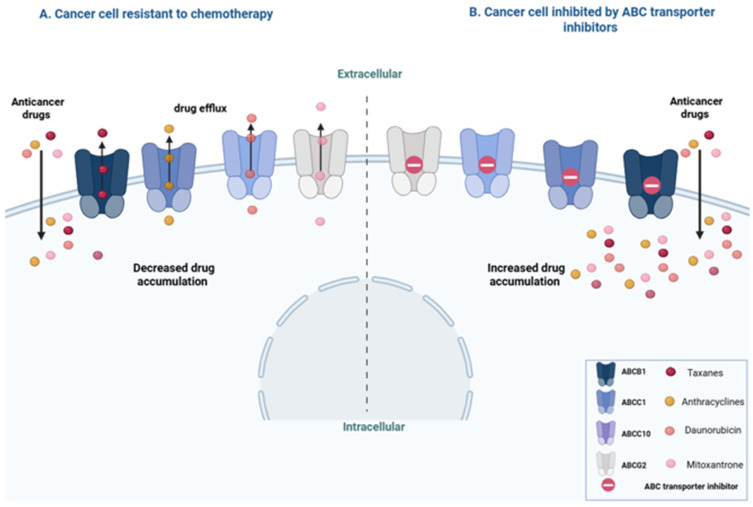
Inhibition of ABC transporters restores chemosensitivity in resistant cancer cells. (**A**) A cancer cell exhibiting resistance to chemotherapy due to overexpression of ABC transporters, which actively efflux chemotherapeutic agents, thereby reducing intracellular drug levels and promoting survival. (**B**) Inhibition of ABC transporter limits drug efflux, reduces drug efflux, and leads to increased intracellular drug concentration and enhanced cancer cell death.

Many investigations have documented the overexpression of P-gp in clinical specimens of different cancer cells. Several research groups have reported that enhanced P-gp expression is often correlated with a poor response to chemotherapy [17,18]. Consequently, clinical trials were initiated to investigate whether P-gp inhibitors could enhance chemotherapy response and improve treatment outcomes by reducing the efflux of drug molecules, thereby increasing the concentration of the drug in cancer cells [19,20]. While the substrates for each transporter and the role of the transporters in reducing the intracellular concentration of the drug are elucidated in laboratory models, their significance in clinical MDR is still under investigation. Despite initial disappointing results, recent evidence indicates that ABC transporters may indeed be involved in clinical MDR in certain contexts. Clinical studies have indicated that the combination of chemotherapy and transporter inhibitors has resensitized the tumor cells to the anticancer drug [21,22]. A new approach is needed to address this issue by reassessing the target biology and developing effective biomarkers. Multiple recent review papers (e.g., [23,24,25]) have provided tables that summarize the transporters in different families, the ligands for each transporter, and other important information about their expression in different types of cancer. Therefore, we attempted to provide a shorter version of this information in a more visual format in Figure 1.

#### 3.1.1. ABCB1/MDR1/P-gp Transporter

The human ABCB1 protein (also known as P-glycoprotein or P-gp), which is encoded by *abcb1* on chromosome 7p21, was the first ABC transporter to be discovered, in 1976 [20,26,27]. P-gp (molecular weight = 170 kDa) serves as a transporter on the apical membrane in various tissues, including liver, kidney, blood–brain barrier, intestines, and, perhaps most importantly, on all hematopoietic progenitor cells, especially those with pluripotent stem cell characteristics. The primary function of P-gp is to safeguard the cell against external harmful molecules [28,29]. Overexpression of P-gp confers resistance to a broad range of chemotherapeutic drugs, e.g., taxanes, vinca alkaloids, epipodophyllotoxins, and anthracyclines (Figure 1) [29,30]. Moreover, P-gp expression levels in cancer cell lines show a negative correlation with paclitaxel sensitivity, suggesting that P-gp overexpression leads to paclitaxel resistance [31,32,33]. Various mechanisms contribute to P-gp overexpression, including gene amplification, increased transcription (e.g., by RGP8.5), variations in translation efficiency, chromosomal rearrangements (generating hybrid genes), and mutations in *abcb1*. Understanding these mechanisms is crucial for overcoming resistance associated with P-gp overexpression [33,34,35]. Many reports provide evidence that clinically validates the role of P-gp in MDR [27,36,37].

Adding a P-gp substrate or inhibitor to chemotherapy (which would compete with the drug molecules for binding to the membrane efflux protein) has been long studied to overcome resistance driven by P-gp expression (Figure 1). However, life-threatening cardiac side effects in clinical trials terminated the trials in their early stages [27]. In 2018, Nanayakkara et al. reported a restoration of response to daunorubicin in multidrug-resistant ovarian cancer by investigational P-gp inhibitors in vitro and in microtumors [38]. A clinical study in patients with anthracycline-resistant metastatic breast cancer showed that the addition of verapamil (a calcium channel blocker that also inhibits P-gp) to vindesine and 5-fluorouracil increased the overall survival (OS; *p* = 0.036) and Progression-Free Survival (PFS; not statistically significant) [39]. Other clinical trials on the next generations of P-gp inhibitors have failed to deliver a similar promise in clinical settings, which could be at least partially explained by the toxic effects of inhibiting a protein with such protective functions [40]. This highlights the potential impact of the targeted delivery of P-gp inhibitors (small molecules, antibodies, or RNA-interfering nucleic acids) on cancer cells. Many nanoparticle-based approaches have been reported in recent years [41].

#### 3.1.2. ABCC1/MRP1 Transporter

Multidrug resistance protein 1 (MRP1), or ABCC1, coded by *abcc1* (on chromosome 16q13), was first identified in the early 1990s [42]. MRP1 (190 Da) is located in the plasma membrane of the liver, kidney, intestine, blood–brain barrier, and placenta [43]. It is involved in the efflux of organic anionic molecules, e.g., sulfates, glucuronides, and other compounds, thereby regulating their cytoplasmic concentrations and influencing the pharmacokinetics of various drugs [44,45]. MRP1 is often overexpressed in MDR cancer cells and contributes to resistance against a wide range of antitumor drugs, e.g., camptothecins, anthracyclines, epipodophyllotoxins, and vinca alkaloids [46]. Several MRP1 inhibitors have been identified, including probenecid, sulfinpyrazone, and indomethacin [47]. Certain inhibitors, such as verapamil, quercetin, and genistein, which primarily target P-gp, also reduce the activity of MRP1. Agosterol A, PAK-104P, imidazothiazoles, and steroid derivatives are among dual inhibitors of P-gp and MRP1 (Figure 1) [48]. However, developing specific and safe MRP1 inhibitors is challenging due to its role as an anionic transporter and to its toxicity (e.g., probenecid) or limited efficacy (e.g., sulindac). Further studies are needed to identify more promising MRP1 inhibitors [49,50]. Additionally, novel strategies such as silencing MRP1 via siRNA could provide a targeted approach to sensitize the cells [51].

#### 3.1.3. ABCC10/MRP7 Transporter

The *abcc10* gene encodes a 171-kDa protein named multidrug resistance-associated protein 7 (MRP7). This protein is universally expressed in various tissues [42,52,53]. It has been proposed that MRP7 has a bipartite substrate-binding pocket that allows interactions with both lipophilic and anionic ligands [54].

Functionally, ABCC10 acts as an active transporter for a wide array of substrates, including chemotherapeutic agents, e.g., antifolates, vinca alkaloids, taxanes, cisplatin, irinotecan, daunorubicin, epothilone B, etoposide, and nucleoside analogs [55]. It also transports cyclic endogenous compounds, nucleotides, exogenous molecules, and their glutathione conjugates [43]. Overexpression of ABCC10 is involved in MDR by reducing the drug concentrations of paclitaxel, docetaxel, and vinca alkaloids inside the cells (Figure 1) [56].

Recent investigations have identified several inhibitors of ABCC10, e.g., sildenafil, cepharanthine, imatinib, nilotinib, erlotinib, vardenafil, and lapatinib, which effectively counteract ABCC10-mediated drug resistance. It has been reported that lapatinib can enhance the response of cancer cells to taxanes by inhibiting ABCC10 activity, while sildenafil and vardenafil restore drug efficacy by blocking ABCC10-mediated efflux (Figure 1). Despite these advances, no clinical studies have confirmed the involvement of ABCC10 in clinical drug resistance, underscoring the need for further investigation in this area [52,54].

#### 3.1.4. ABCG2/BCRP/MXR Transporter

ABCG2, or breast cancer resistance protein (BCRP), or Mitoxantrone Resistance Protein (MXR), is a key efflux transporter that is coded by *abcg2* located on chromosome 4 at position 4q22 [57,58]. Initially identified in 1998 as a protein overexpressed in multidrug-resistant breast cancer cell lines, ABCG2 has a molecular weight of about 72 kDa, which is consistent with the characteristics of ABC transporters [57,59]. ABCG2 is found primarily in the plasma membrane in the liver, brain, small intestine, placenta, ovaries, and prostate [10]. BCRP is involved in the efflux of various compounds, including mitoxantrone. Overexpression of BCRP is reported in mitoxantrone-resistant colon cancer cells, such as S1-M1-80, which results in resistance to mitoxantrone by reducing its intracellular accumulation (Figure 1). Therefore, BCRP is also known as mitoxantrone resistance protein (MXR) [11,60]. Mitoxantrone resistance due to BCRP overexpression is not limited to colon cancer cells; it has also been observed in other cancer cell lines, such as gastric carcinoma, non-small cell lung cancer, breast cancer, myeloma, fibrosarcoma, and glioblastoma [61,62,63]. Additionally, BCRP contributes to resistance against irinotecan-based therapies [64]. A poor clinical response has been reported in patients with metastatic breast cancer, multiple myeloma, and leukemia with overexpression of BCRP [65].

ABCG2 inhibitors are broadly classified into dynamic and static inhibitors. Dynamic inhibitors, such as PZ-39, promote the degradation of ABCG2 molecules as well. In contrast, static inhibitors, including fumitremorgin C (FTC), suppress ABCG2’s function without affecting its stability. Unfortunately, FTC was not developed clinically due to its neurotoxicity [66]. Another potential therapeutic approach is the induction of ABCG2 degradation by xanthine derivatives through lysosome-mediated mechanisms [67]. Furthermore, RNA interference (RNAi), antisense oligonucleotides, or hammerhead ribozymes reversed drug resistance by downregulating ABCG2 expression [68,69,70].

Table 1 presents a comparison of key ABC transporters across several major cancer types, indicating whether their expression levels are increased or decreased and outlining the associated biological effects of these alterations.

### 3.2. DNA Damage Response

The DNA damage response (DDR) contains a sophisticated network of cellular signaling pathways that identify, signal, and repair DNA lesions. This system is important to ensure genomic integrity, particularly in the face of endogenous threats, such as reactive oxygen species generated during cellular metabolism, and exposure to ionizing radiation, ultraviolet light, and chemotherapeutic agents [89]. The DDR utilizes DNA repair mechanisms, cell cycle arrest, and apoptosis to preserve cellular homeostasis, and thereby prevents the accumulation of potentially oncogenic mutations. Important mediators of the DDR are sensor proteins, such as ataxia–telangiectasia mutated (ATM), ATM and Rad3-related (ATR), and DNA-dependent protein kinase (DNA-PK), which trigger responses that coordinate the repair mechanisms [89].

Defects in the DDR pathways are closely linked to tumor development. For instance, germline mutations in DDR-associated genes (e.g., *brca1*, *brca2*, and *tp53*) are well-documented in hereditary cancers, including breast and ovarian malignancies [90]. While impaired DDR function sensitizes the cells to DNA-damaging molecules, it simultaneously drives the emergence of resistance mechanisms. Such adaptive responses include enhanced DNA repair capacity, activation of compensatory pathways, and epigenetic reprogramming [91]. These processes are central to the development of MDR. Persistent activation of DDR pathways plays a pivotal role in MDR, enabling tumor cells to repair therapy-induced DNA damage and survive under selective pressure [90]. Additionally, DDR-related signaling can upregulate efflux transporters and anti-apoptotic proteins, further contributing to the resistance. Targeting DDR components seems to be a promising approach to overcome MDR [90].

#### 3.2.1. Resistance to PARP Inhibitors

**Figure 2:**
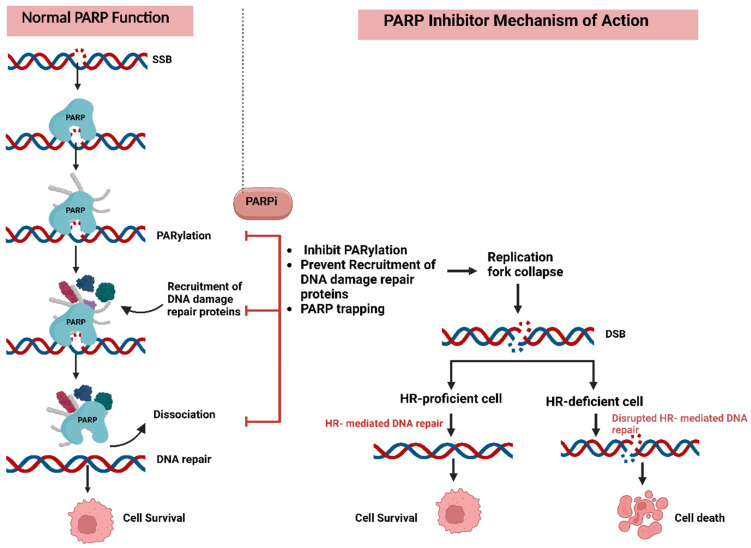
Schematic representation of the mechanism of a PARP inhibitor: Single-strand DNA breaks, which frequently occur in rapidly proliferating cells such as cancer cells, are typically repaired by PARP enzymes to maintain cell survival. PARP inhibitors prevent PARP from binding to DNA damage sites, blocking SSB repair. As a result, the unrepaired lesions can convert into double-strand breaks (DSBs) during DNA replication. In homologous recombination (HR)-proficient cells, DSBs are efficiently repaired, preserving genomic stability. However, in HR-deficient cells, DSBs remain unrepaired, leading to cell death through apoptosis.

Poly (ADP-ribose) polymerase (PARP) enzymes are important molecules in repairing single-strand DNA breaks (SSBs) through the base excision repair pathway [90]. Inhibiting PARP results in the accumulation of double-strand DNA breaks (DSBs) during replication, which are repaired by homologous recombination (HR). Tumors with HR deficiencies, such as *brca1/2* mutations, are particularly vulnerable to PARP inhibitors (PARPis) due to their inability to repair double-stranded breaks, which results in cell death. This “synthetic lethality” phenomenon has been a foundation for the development of PARP inhibitors [90,92]. The first approved PARP inhibitor, olaparib, demonstrated significant efficacy in BRCA-mutated ovarian cancers and laid the basis for the development of other inhibitors (e.g., talazoparib, niraparib, and rucaparib). These agents have shown clinical utility in pancreatic, prostate, and breast cancers (Figure 2) [93].

**Figure 3:**
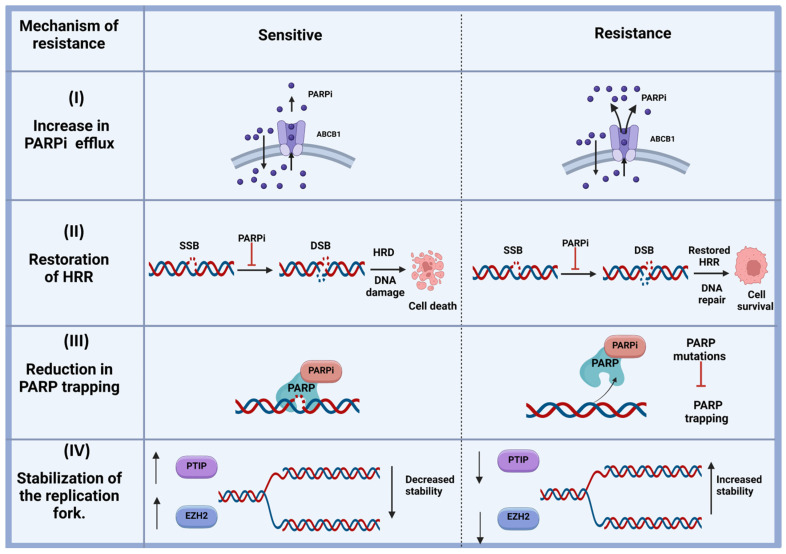
Mechanisms of resistance to PARP inhibitors (PARPis). (I) Increased PARPi efflux: Some PARPis, such as olaparib, are substrates of MDR1 (P-gp). By upregulating the activity of MDR1, these cells can effectively pump the drug out of the cancer cells, contributing to resistance. (II) Restoration of homologous recombination repair (HRR): The concept of synthetic lethality relies on the combination of HRR deficiency and PARP inhibition. When HRR is restored in cancer cells due to factors such as reversion mutation, DNA damage can be repaired through the HRR pathway. This repair allows cancer cells to survive, rendering them resistant to PARP inhibitors. (III) Reduction in PARP trapping: Mutations in PARP may reduce its ability to bind to PARPis, thereby reducing PARP trapping. These mutated forms of PARP can recruit other DNA repair proteins to repair single-strand breaks, resulting in PARPi resistance and enhanced cell survival. (IV) Stabilization of the replication forks: Certain HRR-related proteins also contribute to the stabilization of stalled DNA replication forks. In HRR-deficient cells, which are sensitive to PARPis, stalled replication forks are typically degraded by DNA nucleases (DSB: double-strand break; EZH2: Enhancer of Zeste Homolog 2; HRD: homologous recombination deficiency; HRR: homologous recombination repair; PTIP: Pax2 transcription-interacting protein; SSB: single-strand break).

Resistance to PARP inhibitors can arise through several mechanisms that enable tumor cells to bypass the effects of treatment. A key mechanism is the restoration of homologous recombination repair, often through secondary mutations in HR-related genes (e.g., *brca1* or *brca2*), which restore their functionality and prevent the synthetic lethality induced by PARP inhibition [94]. BRCA1 and BRCA2 are both considered biomarkers for DNA repair capacity [95,96]. Other biomarkers for DNA repair function include the homologous recombination deficiency test, SLFN11, ERCC1, and ATM [97]. Epigenetic modifications also contribute by reactivating promoter methylation in genes such as *brca1* or *rad51c* and thereby reactivating their expression and allowing cells to repair double-stranded DNA breaks via homologous recombination [98]. The stabilization of replication forks is another important factor. For example, the loss of MRE11 activity prevents the degradation of stalled replication forks, helping to maintain genomic integrity [90,93]. Furthermore, reduced PARP1 trapping can diminish the efficacy of PARP inhibitors. This may occur due to *parp1* mutations that impair its ability to bind DNA or increase drug efflux driven by the overexpression of ABCB1 transporters (Figure 3) [90,93].

Efforts to overcome resistance to PARP inhibitors (PARPis) have centered on combination therapies and the exploration of novel targets. Combining PARPis with agents targeting complementary pathways, such as the PI3K/AKT/mTOR axis, induced HR deficiency in preclinical models [93,98]. A combination of the BET inhibitor ZEN-3694 and the PARP inhibitor talazoparib in patients with BRCA1/2 wild-type tumors showed that the objective response rate (ORR) was 22%, and the clinical benefit rate reached up to 35% [99]. Similarly, the addition of antiangiogenic agents like VEGF inhibitors, including bevacizumab, has shown synergistic effects by creating hypoxic conditions and downregulating HR repair mechanisms [100]. Another strategy involves targeting the proteins that play a role in DNA damage response (e.g., ATR, CHK1, and WEE1). This strategy has been promising in counteracting resistance. Additionally, identifying biomarkers such as BRCA reversion mutations, promoter methylation, and immune signatures, can facilitate personalized treatment, improving the selection process of patients who are the best candidates for these therapeutic strategies [101]. In patients with BRCA wild-type and non-HRD ovarian cancer, the combination of niraparib and pembrolizumab showed improved clinical outcomes compared to monotherapy. The dual therapy achieved an ORR of 18% and a disease control rate (DCR) of 65% [102]. Two recent Phase III clinical trials (NCT02000622 and NCT01945775) in metastatic and advanced triple-negative breast cancer have shown increased Progression-Free Survival (PFS) with olaparib and talazoparib, respectively [103]. A recent meta-analysis study revealed that the combination of PARP inhibitors and chemotherapy is only beneficial in patients with the biomarkers for impaired DNA repair function, and even in those patients, the benefits should be weighed against the potential adverse effects [97].

#### 3.2.2. Resistance to Polθ Inhibitors

Coded by the *polq* gene, DNA polymerase theta (Polθ) is integral to DNA repair mechanisms, particularly for double-strand breaks. Polθ plays an important role in the theta-mediated end joining (TMEJ) pathway, which is an alternative to non-homologous end joining (NHEJ) and HR pathways. TMEJ is characterized by its reliance on microhomology sequences during the repair of double-stranded breaks, which sometimes creates errors [104]. Polθ is essential for cell survival in HR-deficient cancers (e.g., those associated with *brca1* or *brca2* mutations) by compensating for the loss of HR-mediated repair [48,104], pointing to Polθ as a potentially promising therapeutic target. Some small-molecule Polθ inhibitors, e.g., novobiocin, selectively target the ATPase domains of polymerases and induce synthetic lethality in HR-deficient tumor models [48]. Nevertheless, resistance to Polθ inhibitors can develop, possibly by upregulation of compensatory DNA repair pathways, such as NHEJ, or by Polθ mutations that do not impair its function but reduce its binding. Additionally, tumors may adapt by restoring HR functionality, which reduces the importance of Polθ in this context [105]. Dual inhibition of both DNA-PK and Polθ, which exploits the synthetic lethality of these pathways, could be effective for overcoming this resistance, especially in p53-deficient tumors [106].

#### 3.2.3. Resistance to ATR Inhibitors

As a serine/threonine kinase, ataxia telangiectasia and Rad3-related protein (ATR) plays an important role in maintaining genomic stability. ATR is a component of the response to DNA replication stress and double-strand breaks, initiating a signaling pathway that regulates DNA repair and cell cycle progression [107,108]. This kinase is activated by the detection of DNA damage or replication obstacles, which leads to phosphorylation of downstream effectors such as checkpoint kinase 1 (CHK1). These events lead to cell cycle arrest and could facilitate DNA repair or apoptosis [107,108].

The potential of ATR inhibition in cancer therapy has also attracted much attention. It has been shown that if DNA repair mechanisms are defective, especially for tumor cells harboring mutations in the *brca1/2* genes, the cells would be sensitive to ATR inhibitors [109]. Several ATR inhibitors have been developed to target cells impaired in DNA repair pathways. Examples include berzosertib (M6620), ceralasertib (AZD6738), and elimusertib [107]. ATR inhibitors (ATRis) leverage synthetic lethality, which leads to selective cell death in the presence of defective DNA repair mechanisms. However, the clinical application of ATRis has encountered challenges, including the emergence of resistance that compromises their effectiveness [109].

Resistance to ATR inhibitors is driven by multiple factors, including a compromised nonsense-mediated decay (NMD) pathway, e.g., UPF2, which enhances tolerance to DNA replication stress and diminishes the effectiveness of ATRis [110]. Additionally, cancer cells may adapt by activating alternative DNA damage response pathways to withstand ATR inhibition. For instance, increased activity of the DNA-dependent protein kinase catalytic subunit has been linked to resistance against DNA repair inhibitors [105]. On the other hand, combined therapies have shown potential. Pairing ATRis with agents like PARP inhibitors leverages synthetic lethality to target cancer cells more effectively [111]. Identifying biomarkers associated with ATRi resistance, such as the loss of NMD components like UPF2, can facilitate the selection of patients and optimization of treatment [110].

Research is ongoing on developing next-generation ATRs with enhanced potency and reduced susceptibility to resistance. The aim of this type of research is to exploit specific genetic vulnerabilities in cancer cells to improve therapeutic outcomes [107]. BAY 1895344 monotherapy demonstrated encouraging antitumor activity, achieving an objective response rate (ORR) of 19% and a disease control rate (DCR) of 57.1% across patients with advanced solid tumors [112].

#### 3.2.4. WRN Inhibitors

Werner syndrome ATP-dependent helicase (WRN) is a member of the RecQ family of DNA helicases that uniquely possesses both helicase and 3′ to 5′ exonuclease activities [113]. Germline mutations in the gene encoding WRN are implicated in maintaining genome stability [114]. By 2019, multiple independent studies showed a lethality relationship between WRN and microsatellite instability (MSI) tumors, sparking growing interest in therapies targeting WRN. Several WRN inhibitors have been developed, and their therapeutic potential is being explored. For example, 2-sulfonyl/sulfonamide pyrimidine derivatives in sulfonamide NH group H3B-960 exhibited an IC₅₀ of 22 nM, and in H3B-968, an IC₅₀ of approximately 10 nM was exhibited [115]. The discovery of effective WRN inhibitors has been challenging. Earlier compounds, such as NSC617145, NSC19630, and ML216, were rigorously tested but failed to demonstrate specific activity against WRN [116]. Inhibitors, such as HRO761 and VVD-133214, work by binding to an allosteric site near C727, causing conformational changes in WRN that improve target selectivity. Currently, both compounds are in Phase I clinical trials [117]. These discoveries emphasize the significant potential of WRN inhibitors for treating MSI cancers.

#### 3.2.5. Resistance to Topoisomerase Inhibitors

DNA topoisomerases are crucial for maintaining the structural integrity of DNA during replication, recombination, transcription, and chromosome segregation [118,119,120]. By creating temporary single- or double-stranded breaks in DNA, they alleviate torsional stress caused by supercoiling and resolve entangled or catenated DNA molecules. Topoisomerases are categorized as follows: type I cleave one strand of DNA and relax supercoiling, whereas type II cleave both strands and facilitate decatenation of intertwined DNA [118,120,121,122]. These enzymes are indispensable for cellular viability, as unresolved topological stress can result in DNA damage and compromised genomic stability [122,123]. However, the covalent enzyme–DNA intermediates formed during the catalysis cycle, if stabilized, are vulnerable to DNA–protein crosslinks (DPCs) and cytotoxicity [123].

Topoisomerases are overexpressed in many cancers because of the elevated proliferation rates, making them attractive targets for anticancer therapies [118,124]. In a clinical study in 57 newly diagnosed acute myeloid leukemias, low topoisomerase IIα predicted lower Progression-Free Survival (PFS; p = 0.03) and lower overall survival (OS; p = 0.03) [125]. Inhibitors of these enzymes act either by stabilizing the transient cleavage complexes (poisons) or by affecting the catalytic activity of the enzyme. For instance, camptothecin and its derivatives, such as irinotecan and topotecan, target topoisomerase I (TOP1), while etoposide and anthracyclines target topoisomerase II (TOP2) [118,124,126,127]. These drugs exploit the natural enzymatic activity of topoisomerases to induce DNA breaks, overwhelming the cell’s repair capacity and triggering apoptosis. Despite their efficacy, these agents often exhibit significant off-target toxicity, as they also affect normal proliferating cells. Despite the initial efficacy of topoisomerase inhibitors, the frequent development of resistance limits their long-term utility in cancer treatment [124,126,127,128,129].

Resistance to topoisomerase inhibitors is multifactorial and is involved in both acquired and intrinsic mechanisms. Topoisomerase gene mutation is a well-known mechanism of resistance to chemotherapy. Point mutations in inhibitors, particularly in active sites or in regions critical for drug interaction, can diminish their binding affinity. For example, *top2* mutations can impair the binding of etoposide and anthracyclines [130,131,132].

Mechanisms other than genetic mutations also contribute to resistance. The expression levels and enzymatic activities of topoisomerases can be changed by epigenetic and post-translational modifications. Furthermore, MDR cells frequently overexpress ABC transporters, (e.g., P-gp). These transporters actively expel topoisomerase inhibitors and reduce intracellular drug concentrations to subtherapeutic levels [133,134]. Interestingly, cancer cells can also upregulate DNA repair pathways, such as the tyrosyl–DNA phosphodiesterase systems (TDP1 and TDP2), by removing stabilized topoisomerase–DNA complexes. This contributes to resistance. Additionally, cancer cells can survive in the presence of topoisomerase inhibitors through metabolic reprogramming and the enhancement of anti-apoptotic signaling [135,136,137].

Proteasomal degradation of trapped enzyme–DNA complexes can also lead to resistance. For instance, topoisomerases can be degraded by nuclear proteases such as SPARTAN and other covalently bound repair proteins. Cancer cells can also compensate by activating HR, NHEJ, and other relevant proteins to repair drug-induced double-stranded DNA breaks. Finally, hypoxia, acidosis, and other factors in the tumor microenvironment can contribute to therapeutic resistance by interfering with drug activity [136,137,138,139].

One of the promising strategies being investigated to counteract resistance to topoisomerase inhibitors is the development of new inhibitors with improved properties, such as the non-camptothecin TOP1 inhibitors, indenoisoquinolines, which have enhanced efficacy [140,141]. Furthermore, drug delivery and retention in tumor tissues have been improved by using PEGylated irinotecan formulations and liposomal encapsulations [142,143,144,145].

Antibody/drug conjugates (ADCs) can be designed to selectively target cancer cells. The conjugate DS-8201, consisting of the HER2-targeting antibody, trastuzumab, linked to a TOP1 inhibitor, has shown promise in clinical studies on HER2-positive cancers [146]. Topoisomerase inhibitors synergized with DNA damage response modulators such as PARP inhibitors in preclinical models. These combinations exploit synthetic lethality in tumors with defects in their DNA repair pathways, such as cancer cells with a mutation in *brca* [93]. Inhibiting drug efflux transporters or targeting the proteasomal degradation pathways of topoisomerase–DNA complexes are also being investigated. The identification of predictive biomarkers, such as TOP1/TOP2 expression levels or specific mutations, may enable personalized treatment that enhances the efficacy of the treatment and reduces the risk of resistance [147,148].

### 3.3. The Role of Apoptosis in MDR

**Figure 4:**
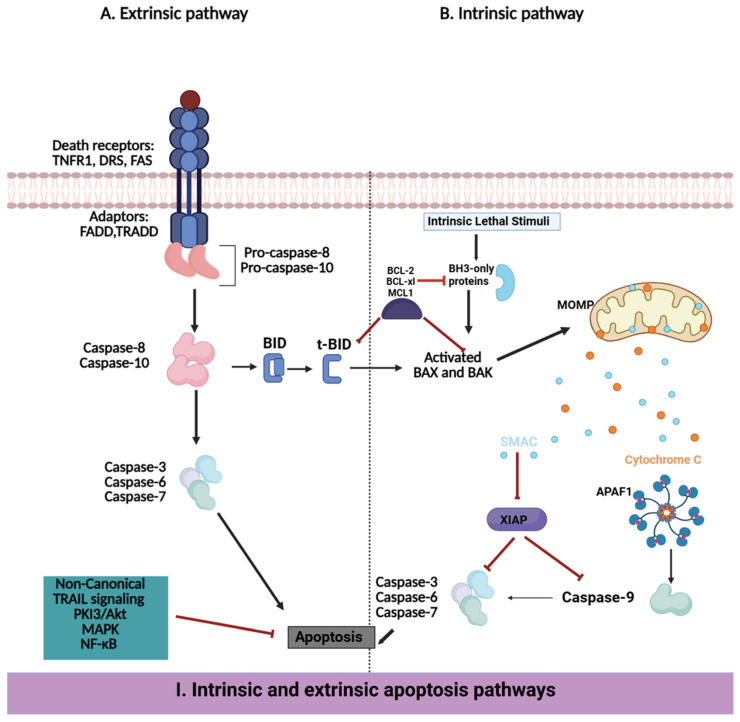

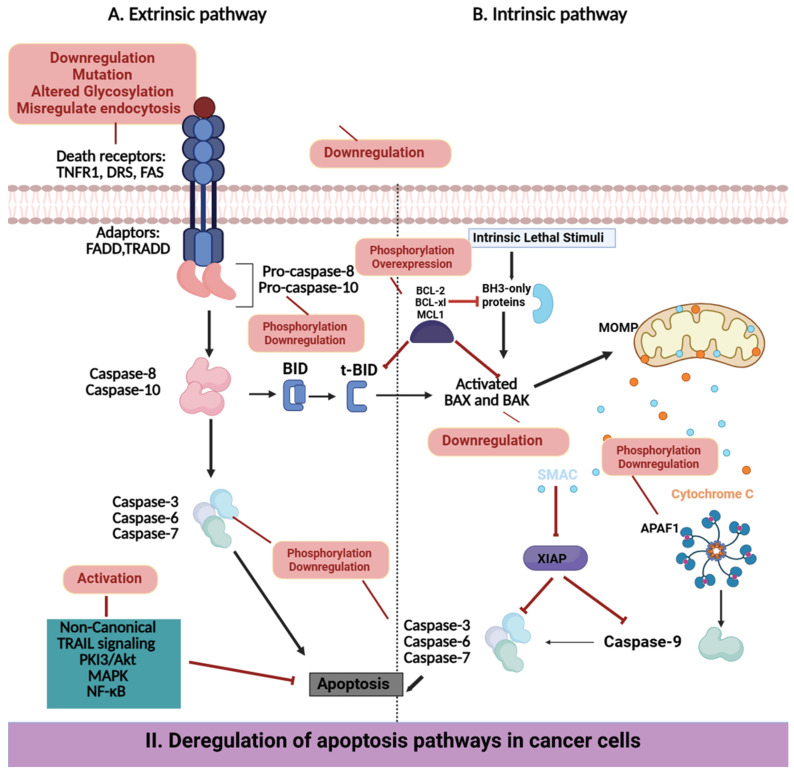
(**I**) Extrinsic and intrinsic apoptosis pathway. External ligands such as TRAIL or FasL bind to death receptors, triggering the recruitment of FADD, which activates initiator caspase-8 and -10. The active forms of caspase-8 and -10 then cleave and activate effector caspases, such as caspase-3, -6, and -7, thereby executing apoptosis. Moreover, caspase-8 cleaves Bid, promoting the oligomerization of Bax and Bak on the outer mitochondrial membrane. This promotes the release of cytochrome c, leading to the activation of caspase-9 and subsequently amplifying the apoptotic signal through further activation of effector caspases. Intrinsic apoptosis can also be initiated by BH3-only proteins, which inhibit the activity of anti-apoptotic proteins like Bcl-2 and Bcl-XL, thereby enabling Bax and Bak to initiate mitochondrial outer membrane permeabilization and cell death. Additionally, inhibitory proteins like XIAP suppress apoptosis by directly preventing the activation of caspases. (**II**) Deregulation of extrinsic and intrinsic apoptosis pathways: The delicate balance between pro-apoptotic and anti-apoptotic signaling molecules is usually disrupted in cancer cells. This deregulation arises from alterations at the transcriptional level, such as abnormal DNA methylation, and at the post-translational level, which together contribute to the evasion of apoptosis.

Apoptosis has a fundamental role in tissue homeostasis by eliminating abnormal or damaged cells. In cancer, disruption of apoptosis mechanisms contributes to unchecked cell proliferation and therapeutic resistance. Apoptosis is regulated through two interrelated pathways: the intrinsic (mitochondrial) pathway and the extrinsic (death receptor) pathway, both of which ultimately activate caspases that disassemble the cells (Figure 4I) [149,150,151]. The B-cell lymphoma 2 (BCL-2) protein family regulates the intrinsic apoptotic pathway, which governs mitochondrial outer membrane permeabilization. Pro-apoptotic proteins (e.g., BAK and BAX) facilitate cytochrome c release, which activates caspase, and anti-apoptotic proteins (e.g., BCL-2 and BCL-XL) counteract this mechanism [151,152,153]. On the other hand, the extrinsic pathway is triggered by the binding of extracellular ligands to death receptors (e.g., Fas and tumor necrosis factor receptor, or TNFR), which leads to the activation of caspase-8 and downstream apoptotic events [152,153,154]. Apoptotic dysregulation in cancer can be generated through various mechanisms, which include loss of pro-apoptotic regulators, overexpression of anti-apoptotic proteins (for example, MCL-1, BCL-2), and defects in death receptor signaling. Such alterations enable cancer cells to evade cell death even in the presence of therapeutic interventions or genomic stress (Figure 4II) [155,156]. In a clinical study in 57 newly diagnosed acute myeloid leukemias, low BCL-2 expression levels predicted longer Progression-Free Survival (PFS) (*p* = 0.02) and longer overall survival (*p* = 0.06) [125].

Therapeutic strategies aimed at restoring apoptosis have gained significant attention in oncology. Several inhibitors targeting anti-apoptotic proteins have been developed: BH3 mimetics like venetoclax, which selectively inhibit BCL-2, have shown clinical efficacy, especially in hematologic malignancies in combination with other drugs like cytarabine, which improved ORR by 54% [157,158]. These agents mimic natural pro-apoptotic BH3-only proteins and displace pro-apoptotic effectors from anti-apoptotic proteins, triggering apoptosis in cancer cells. Venetoclax is approved for the treatment of acute myeloid leukemia and chronic lymphocytic leukemia, in which BCL-2 overexpression plays a critical role in disease progression [159]. Other BH3 mimetics targeting proteins such as BCL-XL or MCL-1 are in clinical evaluation.

MCL-1 inhibitors, including S63845 and AMG 176, have been promising in preclinical studies and early-phase clinical trials for hematologic and solid tumors resistant to BCL-2 inhibition [157,160]. BCL-XL inhibitors, such as A-1331852 and WEHI-539, are being studied in solid tumors in which BCL-XL is a major survival factor [161,162]. Furthermore, mimetics of second mitochondria-derived activator of caspase (SMAC) target inhibitor of apoptosis proteins (IAPs), which often confer resistance to apoptosis. These agents, such as LCL161 and Birinapant, promote the degradation of IAPs, thereby enhancing apoptotic sensitivity and improving treatment outcomes [162,163]. SMAC mimetics have demonstrated efficacy when combined with other therapeutics, predominantly in cancers with overexpression of IAP [163]. Nevertheless, resistance to apoptosis-targeted strategies remains a major impediment. Tumor cells circumvent apoptosis by upregulating anti-apoptotic proteins such as BCL-XL and MCL-1, mutations in pro-apoptotic effectors, or activating compensatory survival pathways, including PI3K/AKT and NF-κB signaling cascades. Counteracting these resistance mechanisms is crucial for improving the efficacy and durability of apoptosis-based treatments [164]. Efforts have focused on approaches that simultaneously target different apoptotic regulators. For example, dual inhibition of MCL-1 and BCL-2 has shown promise in preclinical studies of leukemia and solid tumors [165,166]. Combining BH3 mimetics with inhibitors of compensatory survival pathways, such as PI3K with MEK inhibitors, is under investigation to counteract resistance and enhance apoptosis induction [167]. Incorporating apoptosis-targeting agents with chemotherapy, targeted therapy, or immunotherapy has also been studied as a viable strategy to overcome resistance. Targeting more than one survival pathway simultaneously reduces the chance of developing therapy resistance [168,169]. Moreover, the tumor microenvironment could be modulated to influence the sensitivity to apoptosis. Immune cells, extracellular matrix components, and cancer-associated fibroblasts can contribute to apoptotic resistance by secreting survival-promoting factors.

The efficacy of apoptosis-inducing therapies could be enhanced by targeting these microenvironmental elements with stromal inhibitors, immune checkpoint inhibitors, or cytokine modulators [170,171,172,173]. Reactivation of dormant apoptotic pathways is also being investigated. P53-mediated apoptotic signaling has been reactivated with small-molecule drugs that restore mutant p53 function, such as low-dose APR-246, which significantly enhanced the potency of NAX compounds, reducing their IC₅₀ up to fourfold. Alternatively, autophagy could be inhibited to prevent cancer cells from using other survival mechanisms [174,175].

### 3.4. Glutathione S-Transferases

A family of phase II detoxification enzymes, glutathione S-transferases (GSTs), can catalyze glutathione (GSH) conjugation to various electrophilic and hydrophobic compounds, which leads to an increase in the water solubility of these compounds and expedites their excretion [176]. These enzymes are essential in protecting cells against oxidative stress; however, their overexpression could also lead to drug resistance in tumor cells [177]. The GST family of enzymes is categorized as α, µ, π, Σ, θ, and ζ. The π class (GSTP1) is the most commonly associated with chemoresistance [178].

GSTs can promote resistance to chemotherapy by direct detoxification of chemotherapeutic agents. Many anticancer drugs, such as alkylating agents (e.g., cyclophosphamide, chlorambucil), anthracyclines such as doxorubicin, and platinum-based drugs such as cisplatin, are GST substrates [179,180,181]. These drugs are conjugated with GSH, which results in more water-soluble and less toxic compounds that can be easily transported out of the cell by ABC transporters such as MRP1 and MRP2 [182]. This process reduces the concentration of drug molecules inside the cell and contributes to treatment failure. Beyond their role in drug detoxification, GSTs also influence key signaling pathways that play a role in apoptosis and survival. GSTP1, for example, interacts with c-Jun N-terminal kinase (JNK), a critical regulator of apoptosis [183]. GSTP1 normally forms a complex with JNK, preventing its activation. However, upon exposure to stress signals, this interaction is disrupted, allowing JNK to phosphorylate downstream effectors that induce apoptosis. In cancer cells with strong GSTP1 expression, the sequestration of JNK limits its pro-apoptotic activity, thereby promoting cell survival and resistance to chemotherapy [184].

Moreover, GSTs contribute to drug resistance by modulating intracellular redox homeostasis. The toxic effect of many chemotherapeutic agents is due to the generation of reactive oxygen species (ROS), which induce oxidative stress and trigger cell death. GSTs, and especially alpha and mu, play key roles in neutralizing ROS by facilitating the conjugation of GSH with lipid peroxidation products and other oxidative stress mediators [185]. This protective mechanism enhances the survival of cancer cells due to a lack of response to ROS-inducing drugs, further reinforcing resistance to therapy.

The overexpression of GSTs in drug-resistant tumors has triggered studies on developing GST inhibitors that can act as chemosensitizers. The ability of several small-molecule inhibitors, such as ethacrynic acid and TLK199, to inhibit GST activity and restore drug sensitivity has been investigated [186]. These inhibitors can be competitors for binding to the active site of GSTs, therefore preventing the conjugation of GSH to chemotherapeutic agents. Combining GST inhibitors with standard chemotherapy was promising in reversing resistance and enhancing drug effectiveness in preclinical studies [187]. Furthermore, gene silencing, such as by CRISPR-Cas9 or effectors of RNAi delivery, has been suggested as a way to selectively downregulate GST expression in resistant cancer cells [184]. Recent advances have paved the way to targeting GST-mediated resistance. Biomarker-driven approaches to assess GST expression levels in tumors may help identify patients who might be candidates for GST inhibitors or other therapeutic strategies. Also, the development of GST-activated drugs is a novel way to utilize the high GST activity in resistant tumors. These prodrugs are inactive in normal tissues but are activated by GST in tumor cells [178].

## 4. Resistance to Molecularly Targeted Therapy

**Figure 5:**
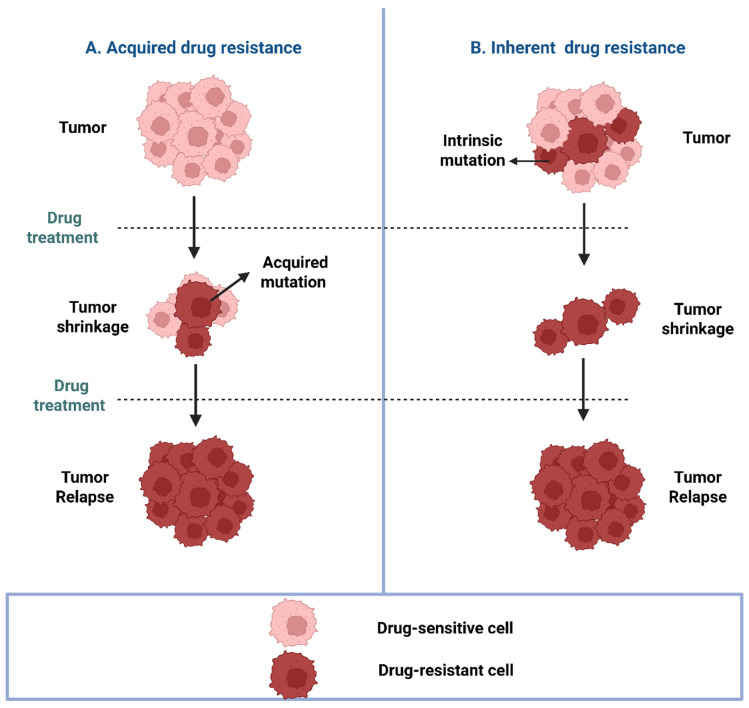
A schematic representation of inherent and acquired drug resistance: In inherent resistance, tumor cells are inherently resistant due to pre-existing mutations. Conversely, acquired resistance arises when tumor cells develop mutations in drug targets during or following therapeutic exposure.

Cancer is a heterogenous disease [188], and its heterogeneity increases as it progresses [189]. This intra- and inter-population heterogeneity results in different expression levels or functionality of proteins involved in the intracellular pathways, creating a subpopulation of cells that do not respond to a previously unused anticancer drug [190]. This “almost Darwinian” model [191] suggests a similar “survival of the fittest” phenomenon, where a subpopulation of cells that are inherently resistant to the effect of the treatment would outlive the other cells and create a platform for the relapse of the tumor. The reemergence of the Cancer Stem Cell Theory in the 1990s [192] linked the idea of differentiation of stem cells into different cancer cells to the existence of an inherently highly resistant subpopulation of stem cells in the tumor that are in charge of metastasis and/or recurrence of the tumor [193]. By definition, stem cells are less likely to undergo apoptosis or DNA damage and could be expected to overexpress cell membrane efflux proteins against exogenous molecules as well [194,195,196,197]. However, inherent resistance is not exclusive to MDR but is also observed against molecularly targeted drugs. The inherent resistance (unresponsiveness of a subpopulation of cancer cells) can be explained by the heterogeneity of malignant cells (Figure 5). A “Big Bang” model is proposed for the tumorigenesis process, suggesting that following the initial mutation that causes oncogenesis, future generations undergo more mutations in separate populations, which leads to “spatial heterogeneity” [15]. This heterogeneity explains over- or under-activation of specific biomarkers and signaling pathways, which leads to a subpopulation of cells that are inherently non-responsive to specific molecularly targeted drugs because they do not rely on the targeted protein for their survival and/or proliferation.

Alternatively, responsive cells can become non-responsive to anticancer drugs over time (acquired resistance), which can develop against molecularly targeted drugs as well as chemotherapy. While mutation in the targeted protein is a major mechanism of resistance development, the underlying mechanisms of acquired resistance could be much more complicated (Figure 5). Whereas plasticity in normal cells is involved in cell differentiation and adaptation to external factors [198], abnormal plasticity in cancer cells [199] is involved in oncogenesis and progression of tumors [200]. However, plasticity is also strongly linked to acquired resistance. In chemotherapy, an endothelial-to-mesenchymal transition (EMT) is induced in response to different chemotherapeutics [201,202], increasing cell mobility and invasiveness and reducing the response to chemotherapy via different mechanisms, including enhancement of DNA repair and efflux protein activity, and the inhibition of the response to pro-apoptotic mechanisms [201,203,204,205]. The EMT can also change the response to immunotherapy by increasing the expression of IL-10 and TGF-β and the overexpression of immune checkpoints [206,207]. However, plasticity contributes to acquired resistance to molecularly targeted drugs by enabling cancer cells to activate other signaling cascades that make up for the loss of action of the targeted molecule. For example, activation of the JAK1/STAT3 pathway in cells resistant to selumetinib (MEK inhibitor), erlotinib (EGFR inhibitor), or crizotinib (c-MET and ALK inhibitor) is another example of plasticity-induced resistance by activation of alternative pathways [208].

Molecular targeted therapies function by targeting a specific molecule in different ways. In addition to affecting proliferation rate, apoptosis, metastasis, angiogenesis, and/or resistance, they could also enhance antitumor immune reaction by recruiting CD8+ T-cells, increasing the cytotoxicity of natural killer cells, inducing death in immunogenic cells, and/or downregulating immunosuppressive myeloid cells [209]. Monoclonal antibodies facilitate immune-mediated cytotoxicity by bridging the tumor and immune system via Fab- and Fc-mediated interactions, which cause opsonization and, subsequently, antibody-dependent cellular cytotoxicity [210]. Moreover, neutrophil-mediated trogoptosis, complement activation, and cytokine regulation further contribute to immune-enhanced tumor cell elimination [211,212]. Despite their efficacy, resistance against molecularly targeted drugs remains a perilous challenge.

In many cases, the molecularly targeted drug is only effective if the targeted molecule is overexpressed and/or overactive in the tumor cells, which highlights the importance of precision medicine practice, where the patients are stratified based on the biomarkers of the activity of the targeted molecule. For example, a recent review paper has evaluated the biomarkers that could indicate the benefits of CDK inhibitors in HR+/HER2- breast cancer patients [213]. This review specifies biomarkers involved in inherent resistance (e.g., cyclin E1, among others) versus the biomarkers of acquired resistance (e.g., AURKA, among others). However, according to the authors, “… no molecular biomarker has satisfied the analytical/clinical validity and clinical utility requirements for its implementation in clinical practice so far” [213].

Genome-wide screening with clustered regularly interspaced short palindromic repeats (CRISPR)/CRISPR-associated nuclease 9 (Cas9), single-cell sequencing, and proteomics are among other approaches that have been recently used for the identification of proteins involved in resistance as potential targets for molecularly targeted drugs. In CRISPR/Cas9 screening, libraries of sgRNAs are used to target and knock out different genes to identify the genes that play a role in resistance against a specific drug. This approach was recently reviewed, focusing on MAPK inhibitors, PARP inhibitors, and other specific pathways and mechanisms [214]. The main advantage of single-cell RNA sequencing is the important information provided about the heterogeneity in a tumor population and the possibility of characterizing diverse populations in a single tumor. Multiple recent reviews have reported the use of this strategy in discovering diverse resistance mechanisms in different types of cancer [215,216,217,218]. Mostly done by mass spectroscopy, among other methodologies, proteomics is used to study the proteins involved in resistance mechanisms and their interactions in complexes [219].

Several next-generation molecularly targeted drugs, including lorlatinib, osimertinib, and sotorasib, have demonstrated substantial clinical benefit, particularly in tumors with specific oncogenic drivers. Improved blood–brain barrier penetration by drugs like lorlatinib and osimertinib has addressed previous therapeutic gaps, especially in metastatic NSCLC. Nonetheless, resistance frequently emerges through secondary mutations (e.g., EGFR C797S, ALK G1202R), compensatory pathway activation (e.g., MET or PI3K upregulation), and bypass signaling. While combination approaches such as BRAF plus MEK inhibition have shown improved efficacy, they can also facilitate the evolution of new resistance routes. Notably, Src-family kinases remain underexploited due to the lack of selective inhibitors, and persistent pathway crosstalk in RAS and JAK signaling limits the durability of responses. These observations highlight the necessity for dynamic and combinatorial treatment strategies to overcome resistance and optimize patient outcomes.

To address MDR, predictive biomarkers that can anticipate therapeutic resistance have become increasingly important in guiding clinical decisions and optimizing personalized treatment strategies. Table 2 summarizes key cancer types, their primary molecular targets, therapeutic options, and well-characterized resistance mechanisms. Additionally, it highlights clinically validated predictive biomarkers currently used to detect or predict resistance to these therapies, enabling timely adjustments in treatment plans and improving patient outcomes.

## 5. Strategies to Overcome Multidrug Resistance in Cancer

### 5.1. Nanotechnology-Based Drug Delivery Systems

Nanotechnology-based drug delivery systems offer innovative approaches that can enhance the efficacy of anticancer drugs by tumor-specific accumulation (via passive and/or active targeting), which can also reduce systemic toxicity [287]. Liposomal formulations such as non-pegylated liposomal doxorubicin (Myocet), pegylated liposomal doxorubicin (Doxil/Caelyx), pegylated liposomal irinotecan (Onivyde), and liposomal daunorubicin (DaunoXome), can increase tumor concentration of the drug [288]. Albumin-bound paclitaxel nanoparticles (Nab-paclitaxel or Abraxane) are another example of this approach [289]. However, there are also reports on the efficacy of this approach in overcoming MDR, mainly by escaping efflux proteins. Many nanoparticles are internalized into target cells by endocytosis, and therefore, theoretically, they can bypass the effect of efflux proteins. Polymeric micelles (e.g., PLGA [290] and Pluronics [291]), dendrimers [292], mesoporous silica nanoparticles (MSNs) [293], and nanostructured lipid–dextran sulfate hybrid carriers (NLDCs) [294] are examples of efforts to overcome MDR using nanoparticles as delivery systems for chemotherapy that have achieved different degrees of success. Co-encapsulation of a chemotherapeutic agent and a P-gp inhibitor has also been studied for liposomal delivery of doxorubicin and verapamil [295], micellar delivery of paclitaxel and verapamil [296], and co-delivery of paclitaxel and tetrandrine by MSNs [297].

### 5.2. RNA Interference and CRISPR/Cas9 Technology

Protein silencing by delivery of siRNA, small hairpin RNA (shRNA), microRNA (miRNA), or clustered regularly interspaced short palindromic repeats (CRISPR)/ CRISPR-associated protein 9 (Cas9) gene-editing systems has been studied as a promising tool to overcome MDR by selectively targeting key genetic determinants of drug resistance. The use of siRNA for overcoming MDR has been studied extensively by targeting molecules involved in enhanced survival, DNA repair mechanisms, and efflux proteins [298,299]. One of the best-documented applications of CRISPR/Cas9 in overcoming MDR is targeting ABC transporters, particularly ABCB1 (P-glycoprotein) and MDR1 [300,301]. Studies have demonstrated that CRISPR/Cas9-mediated knockout of these transporters significantly restores drug accumulation and enhances chemosensitivity in different cancer types, e.g., colorectal and ovarian cancers [302].

Beyond drug efflux, alterations in DNA damage response (DDR) and repair mechanisms are essential in MDR. Overexpression of DNA repair proteins, such as BRCA1, PARP1, and RECQL4, enables cancer cells to repair DNA damage induced by chemotherapy, diminishing the effect of treatments like platinum-based drugs and PARP inhibitors. CRISPR/Cas9 targeting of these genes has been promising in sensitizing the cells to DNA-damaging drugs by impairing HR and NHEJ mechanisms [303].

Mutations resulting in overactivation of oncogenes such as EGFR, BCR-ABL, or KRAS and/or mutations leading to the downregulation of tumor suppressor genes such as *tp53* and *rb1* are frequently associated with MDR. CRISPR/Cas9 technology has been successfully used to disrupt mutant KRAS in colorectal cancer, restoring sensitivity to EGFR inhibitors [304]. Similarly, TP53 knockout in osteosarcoma cells is reported to sensitize the cells to doxorubicin, underscoring the potential of gene editing in modulating key signaling pathways involved in drug resistance [305].

Cancer stem cells are essential for MDR by maintaining self-renewal properties and resisting conventional therapies. Targeting genes such as *cd44* with CRISPR/Cas9 has effectively reduced stem cell populations and enhanced chemosensitivity. Targeting *cd44* in osteosarcoma and hepatocellular carcinoma has been reported to sensitize the cells to doxorubicin and sorafenib, respectively [306].

In addition to direct gene editing, CRISPR/Cas9 screens have been instrumental in identifying novel MDR-associated genes. Genome-wide CRISPR knockout screens have revealed critical regulators of drug resistance, such as SLFN11 (involved in S-phase arrest). Functional validation of these targets has opened new avenues for therapeutic intervention in multidrug-resistant cancers [307,308,309]. While CRISPR/Cas9 holds high hopes for overcoming MDR, several challenges must be addressed before a clinical application. These include limitations in delivery systems’ efficiency, off-target effects, and potential immune responses against CRISPR components. Advances in base editing, prime editing, and nanoparticle-mediated CRISPR delivery are expected to enhance precision and safety, bringing CRISPR-based therapies closer to clinical translation [310,311,312].

### 5.3. Natural Modulators

Natural chemical compounds have emerged as promising for bypassing MDR owing to their potency, low toxicity, and selectivity. These compounds are increasingly recognized as the fourth generation of inhibitors of efflux proteins. Several biologically active natural products extracted from plants and fungi have demonstrated efficacy in reversing MDR by targeting ABC transporters. Curcumin is one of the best-studied natural modulators; it resensitizes MDR cells, but its clinical application is limited by poor bioavailability and rapid metabolism [313]. Likewise, flavonoids, including flavanols, flavones, and isoflavones, act as modulators of ABC transporters, influencing drug absorption, distribution, and elimination. Notably, some flavonoids can either inhibit or stimulate ABC transporters, depending on the specific substrate [314]. The mechanism of action of these natural modulators primarily involves competitive binding to ABC transporters [315].

### 5.4. Physical Approaches

Thermal, ultrasonic, and photodynamic therapies represent key physical strategies for improving the delivery and/or efficacy of chemotherapeutic agents in cancer treatment. Hyperthermia (41–47 °C) is widely recognized for its ability to improve therapeutic outcomes when combined with other treatments [316]. Thermosensitive nanocarriers exhibit phase transitions that facilitate a controlled drug release. Moreover, hyperthermia directly induces tumor cell death by protein denaturation, DNA damage, apoptosis activation, and modulation of the tumor microenvironment [317]. It also mitigates MDR by inactivating detoxification mechanisms, increasing drug uptake, and reducing multidrug-resistant protein expression [318].

Ultrasound therapy has also gained attention for its role in enhancing drug diffusion, improving nanocarrier-mediated drug delivery, and reversing MDR. Ultrasound-responsive nanocarriers facilitate site-specific drug release, which could cause the production of reactive oxygen species, DNA damage, and suppression of MDR-related proteins [319]. Studies demonstrate the effectiveness of co-delivering chemotherapeutics and genetic material, such as siRNA, to improve treatment efficacy [320].

Photodynamic therapy (PDT) employs a photosensitizer, light (600–800 nm), and oxygen to generate cytotoxic reactive oxygen species, leading to targeted cancer cell destruction. PDT contributes to MDR reversal by triggering apoptosis, damaging resistance-associated proteins, and enhancing drug penetration. These physical approaches provide promising adjunct strategies to improve cancer therapy by overcoming resistance mechanisms and optimizing drug delivery [292].

### 5.5. Immunotherapy

The role of the epithelial-to-mesenchymal transition (EMT) in MDR is well accepted. This transition is known to create features that resemble cancer stem cells, including overactivation of signaling pathways involved in enhanced survival, anti-apoptosis mechanisms, and overexpression of drug efflux proteins [321]. One of the other changes associated with the EMT is the overexpression of PD-L1 on the tumor cells [322,323]. The interaction of PD-L1 and PD-1 (on T-cells), as two of the most studied immune checkpoints, has been a popular target for immunotherapy, and, therefore, immunotherapy has been explored as another approach to overcoming MDR [324]. It has even been reported that inhibition of PD-L1 and PD-1 interaction could inhibit P-gp expression [325].

## 6. Conclusions

To answer the titular question: yes, as chemotherapy remains indispensable in cancer therapy, we are still deeply affected by MDR. The challenges due to MDR might be even more significant in developing countries, where access to the most recently developed treatments and diagnostic tools might be limited, which means fewer options are available for treatment when dealing with a potentially more developed tumor. Furthermore, while “off-target” mechanisms involved in resistance against molecularly targeted drugs are significantly more complicated and still relatively unexplored, the fundamentals of MDR mechanisms are similar to some of the mechanisms involved in resistance to molecularly targeted drugs (e.g., modifications to apoptosis pathways). Therefore, strategies to overcome MDR can potentially pave the way to more efficient approaches to overcoming resistance. However, the strategies investigated so far have not been overly promising.

Nanotechnology-based approaches are still not commonly used in clinics, which is likely due to a less-than-optimal performance in vivo, despite promising results in vitro. Also, while encapsulating a molecularly targeted drug in a nanoparticle might enhance tumor accumulation by passive and perhaps active targeting, it is not anticipated to have a significant impact on resistance against the drug (the effect of nanoparticles on MDR is mostly due to escape from efflux proteins). Natural molecules and physical approaches have been around for a while, and even though in some cases, they have shown promise as an alternative approach, they have not made a lasting difference in the overall landscape of cancer therapy. RNAi approaches or CRISPR/Cas9 gene editing look very promising on paper. Primary and secondary mutations that play a significant role in resistance to cancer therapy can easily be nullified by designing nucleic acid sequences that avoid the known mutation sites. Unfortunately, the efficient delivery of nucleic acids to targeted cells has been a major hurdle and has marred the widespread use of these approaches in clinics. Additionally, the belief among clinicians that RNAi is an in vitro approach and has no chance of ever being efficient in clinics is a stigma that is difficult to overcome. With the success of mRNA delivery for vaccination against COVID-19, the development of lipid nanoparticles might change that belief over time. On the other hand, the concern with off-target mechanisms of resistance against molecularly targeted drugs (overactivation of compensatory cellular mechanisms) would not be addressed by using nucleic acids, as we have previously demonstrated in vitro [326].

Combination therapy has been the common practice in cancer treatment for a long time. There is little evidence for synergistic effects among chemotherapeutic agents [327] (discussing flaws in experimental approaches to investigate potential synergy for any drug combination requires another review paper) or for any effect of these combinations on MDR. However, combining drugs from different families of chemotherapeutic drugs with different mechanisms of action, especially if they are not equally affected by the same mechanism of MDR, could offer some benefits in enhancing the efficiency of treatment. On the other hand, the introduction of molecularly targeted drugs to clinics created exciting opportunities for targeting off-target mechanisms of resistance by inhibiting a protein involved in a compensatory pathway(s). However, the recent trend in clinical trials is mostly a combination of chemotherapy and molecularly targeted drugs, as indicated in our recent review of the molecular targets in breast cancer [328]. While this type of combination might enhance the efficiency of treatment slightly (due to additive effects), it is unlikely to have a real impact on resistance. Therefore, at least seemingly, the combinations under investigation are rather arbitrary and not based on the observations made on off-target mechanisms involved in resistance against molecularly targeted drugs. Inhibition (or silencing) of a compensatory mechanism could be a very effective tool in overcoming resistance to molecularly targeted drugs [190], which seems like a wasted opportunity at the moment.

After years of investigation into resistance against cancer therapy, we do not seem to have made satisfactory progress. The most promising options seem to be nucleic acids and combinations of molecularly targeted drugs carefully selected to target the potential off-target mechanism(s). Investing in research focused on improving delivery systems for nucleic acids can bring more RNA- and CRISPR-based medications to clinics and set in motion an approach that is not easily affected by primary or secondary mutations. Benefit would be derived from a more comprehensive investigation of crosstalk among signaling pathways that would lead to the identification of novel proteins that are essential in compensatory mechanisms that create resistance to molecularly targeted drugs. Simultaneous targeting of the selected proteins and the potential compensatory mechanisms can significantly improve the chances of overcoming resistance.

## Figures and Tables

**Table 1:** ABC transporters across different cancer types. Arrows indicate over- or under-expression.

Cancer Type	ABC Transporter(s)	Expression (↑/↓)	Tumor Behavior/Outcome	Remarks
**Neuroblastoma**	ABCC4	↑	Linked to MYCN amplification, advanced stage, lower event-free and overall survival	Independent prognostic marker after adjusting for age, stage, and MYCN [71].
**Glioblastoma**	ABCB1, ABCC1	ABCB1 ↓ in high grade ABCC1 ↑ in high grade	ABCC1 highly expressed in grade III–IV gliomas; associated with malignancy	ABCC1 may indicate undifferentiated glial phenotypes in glioblastoma [72,73,74,75].
**Breast Cancer**	ABCC11, ABCC1, ABCC8, ABCF1	ABCC11 ↑, ABCC1 ↑, ABCC8 ↓	ABCC11 linked to aggressive subtypes (TNBC, HER2+); ABCC8 ↓ expression in higher grades	ABCC11 polymorphism (538G) associated with breast cancer in some populations [76,77,78,79,80,81].
**Prostate Cancer**	ABCB1, ABCA1	ABCB1 ↓ (also ↑ in some cases), ABCA1 ↓	ABCB1 ↓ leads to androgen accumulation and tumor growth; ABCA1 ↓ leads to AKT pathway activation	ABCB1 expression varies by subtype; ABCA1 loss contributes to cholesterol-driven tumor progression [82,83,84,85].
**Ovarian Cancer**	ABCA1, ABCA4	↑	ABCA1 linked to reduced survival; ABCA4 ↑ in stage I epithelial ovarian cancer	ABCA1 inhibition reduces migration and proliferation in vitro [86,87].
**Non-Small Cell Lung Cancer (NSCLC)**	ABCB1, ABCG2	Differential: ↑ in adenocarcinoma (AC)/↓ in squamous cell carcinoma (SCC)	Higher expression in AC contributes to chemoresistance via increased efflux; cisplatin upregulates both in AC and SCC through beta-catenin activation	Wnt microenvironment dictates transporter expression: Wnt7b (canonical) ↑ ABC transporters in AC; Wnt5a (non-canonical) suppresses them in SCC. Cisplatin induces beta-catenin pathway, upregulating ABCB1 and ABCG2 even in SCC [88].

**Table 2:** Selected classes of molecularly targeted inhibitors and their targets, key molecularly targeted drugs, cancer types, mechanisms of resistance, and biomarkers.

Inhibitor Class	Targets	Key Agents	Cancer Types	Resistance Mechanisms	Biomarkers
ALK Inhibitors [220,221]	ALK	Crizotinib [221], Ceritinib, Alectinib [222], Brigatinib, Lorlatinib	NSCLC [223], neuroblastoma, lymphoma	CNS penetration limits (crizotinib), secondary ALK mutations	ALK mutations (e.g., G1202R) [224]
TRK/FLT3 Inhibitors [225,226]	TRKA/B/C, FLT3	Larotrectinib, Entrectinib, Midostaurin	Sarcomas, breast cancer, AML	NTRK mutations (e.g., solvent-front) [227], PTEN loss [228], FLT3-ITD mutations [229]	FL3 mutations [230]
EGFR-Family Inhibitors [231,232,233]	EGFR, HER2, HER3, HER4	Erlotinib, Gefitinib, Osimertinib, Mobocertinib, Trastuzumab	NSCLC, glioblastoma, breast cancer	T790M [234,235], C797S [236], bypass signaling EGFR exon 20 insertions, HER2 amplifications, downstream mutations (PIK3CA, AKT), EMT [237], SCLC transformation [238], BIM loss, PD-L1 overexpression [239]	EGFR T790M (for resistance to 1st/2nd gen TKIs → guides osimertinib use) [240]
MET Inhibitors [241]	MET	Tepotinib, Capmatinib [242]	NSCLC [242]	PI3K/AKT, RAS/RAF pathway overactivation [243]	MET dysregulation (e.g., exon 14 skipping, amplification) [244]
PI3K/mTOR Inhibitors [245]	PI3K isoforms, mTORC1/2	Alpelisib, Copanlisib, Temsirolimus, Everolimus	Lymphoma, breast cancer	Feedback loops [246], ERK/MAPK crosstalk [247]	Positive pS6rp staining combined with KRAS mutation [248]
RAS/RAF/MEK Inhibitors	KRAS [249], BRAF [250], MEK [251]	Sotorasib [252], Dabrafenib, Trametinib [253]	Melanoma, colorectal, thyroid cancer	Pathway reactivation, compensatory signaling [254,255,256]	Lack of expression of DUSP6 [257], mutations in NRAS, RAC1, MAP2K1, MAP2K2, and NF1 [258]
CDK Inhibitors [259,260]	CDK4/6	Abemaciclib, Ribociclib, Palbociclib [261]	Breast cancer	In-target mutations, off-target activation [262,263,264]	Biomarkers of inherent resistance (e.g., cyclin E1, Rb1) and acquired resistance (e.g., AURKA) [213]
JAK Inhibitors	JAK1/2/3, TYK2 [265,266]	Ruxolitinib, Fedratinib [267]	Myeloproliferative neoplasms [268]	Crosstalk with PI3K/AKT/MAPK, secondary mutations [269]	Secondary mutations on Jak2 [269]
BCR-ABL Inhibitors	BCR-ABL1 [270]	Imatinib, Nilotinib, Bosutinib [271], Ponatinib [272], Asciminib [273]	CML, ALL	T315I mutation [274], compound mutations [275], compensatory activation	IL6R, IL7R, and MYC expression [276], miRNAs [277]
SFK Inhibitors	Src-family kinases	Dasatinib (Broad Spectrum, Not Specific [278])	Various	Lack of specific inhibitors, compensatory activation [279]	STAT3 overactivation [280]
Angiogenesis Inhibitors	VEGFR, PDGFR, FGFR [281,282,283], RET [284]	Sorafenib, Sunitinib, Lenvatinib, Pazopanib	Renal, thyroid, ovarian cancer	Common resistance as with other TKIs [285]	Circulating endothelial progenitor cells (CEP)/circulating endothelial cells (CEC) populations, proangiogenic cytokines and tumor endothelial markers (TEMs) [286]

## Data Availability

No new data were created or analyzed in this study. Data sharing is not applicable to this article.
